# Prognostic Value of EMT-inducing Transcription Factors (EMT-TFs) in Metastatic Breast Cancer: A Systematic Review and Meta-analysis

**DOI:** 10.1038/srep28587

**Published:** 2016-06-23

**Authors:** Saber Imani, Hossein Hosseinifard, Jingliang Cheng, Chunli Wei, Junjiang Fu

**Affiliations:** 1 Key Laboratory of Epigenetics and Oncology, the Research Center for Precision Medicine, Southwest Medical University, Luzhou, Sichuan 646000, P.R. China; 2 Chemical Injuries Research Center, Baqiyatallah University of Medical Sciences, Tehran, Iran; 3 Department of Biostatistics, Faculty of Paramedical Sciences, Shahid Beheshti University of Medical Sciences, Tehran, Iran; 4 State Key Laboratory of Quality Research in Chinese Medicine, Macau University of Science and Technology, Macau (SAR), 999078, P.R. China

## Abstract

The epithelial-to-mesenchymal transition (EMT) is a vital control point in metastatic breast cancer (MBC). TWIST1, SNAIL1, SLUG, and ZEB1, as key EMT-inducing transcription factors (EMT-TFs), are involved in MBC through different signaling cascades. This updated meta-analysis was conducted to assess the correlation between the expression of EMT-TFs and prognostic value in MBC patients. A total of 3,218 MBC patients from fourteen eligible studies were evaluated. The pooled hazard ratios (HR) for EMT-TFs suggested that high EMT-TF expression was significantly associated with poor prognosis in MBC patients (HRs = 1.72; 95% confidence intervals (CIs) = 1.53–1.93; P = 0.001). In addition, the overexpression of SLUG was the most impactful on the risk of MBC compared with TWIST1 and SNAIL1, which sponsored fixed models. Strikingly, the increased risk of MBC was less associated with ZEB1 expression. However, the EMT-TF expression levels significantly increased the risk of MBC in the Asian population (HR = 2.11, 95% CI = 1.70–2.62) without any publication bias (t = 1.70, P = 0.11). These findings suggest that the overexpression of potentially TWIST1, SNAIL1 and especially SLUG play a key role in the aggregation of MBC treatment as well as in the improvement of follow-up plans in Asian MBC patients.

Metastatic breast cancer (MBC) is one of the deadliest types of breast cancers (BCs) in females worldwide with a mortality rate greater than 2.1 per million cases yearly^1^,^2^. A multi-step process is involved during the development of metastasis, including local invasion, intravasation, systemic transport, extravasation, and colonization. Therefore, cancer cells are spread through the circulation and finally outgrow to produce macrometastases at distant organs^3^,^4^. The epithelial-to-mesenchymal transition (EMT) is a critical process for the acceleration of non-invasive to invasive breast cancer (IBC) and the increased resistance to conventional chemotherapy, in which polarized epithelial cells lose their tight cell-cell junctions, leading to the enhancement of the migratory capacity and gain in the incursive properties to become mesenchymal cells. EMT is characterized by alterations in gene expression, changes in cellular polarity, disruption of tight junctions, the greatly increased production of extracellular matrix (ECM) components, the production of metalloproteinases (MMPs) and transforming growth factor-β (TGF-β), the expression of cancer stem cell markers, hypoxia, decreased E-cadherin expression, and other molecular biological events^5^,^6^.

The understanding of the mechanistic functions of the EMT markers that have been associated with metastases, as well as their regulation, is thought to be critical to access the potential treatment strategies for MBC patients^7^,^8^. Recent studies have indicated that the EMT is hallmarked by the loss of the cell-cell adhesion molecule E-cadherin, upregulation of more plastic mesenchymal proteins such as Vimentin, MMPs, and N-cadherin, and deregulation of the Wnt pathway, leading to epithelial cells breaking through the basement membrane^9^,^10^. Informatively, the molecular and morphological changes in the EMT are characterized and classified into functional categories: extracellular proteins, cell surface molecules, cytoplasm and nucleolus mediate (Fig. 1 of ref. 11).

Many transcriptional repressors of E-cadherin have been identified, and these included members of the SNAIL1, basic helix–loop–helix (bHLH) families, and double zinc finger E-box binding (ZEB) transcription factors. Among these, nucleolar EMT-inducing transcription factors (EMT-TFs) such as TWIST1, SLUG, SNAIL1, ZEB1 (TCF8/dEF1), ZEB2 (SIP1), E2-2 (TCF4), E47 (TCF3), FOX family and GATA4/6 are often used as biomarkers of EMT^12^,^13^,^14^,^15^.

Many recent studies have tried to determine whether EMT expression may be a prognostic factor for survival in MBC patients. It is interesting to note that aberrant EMT-TFs expression has been widely reported to be related to poor survival and prognosis of MBC^16^,^17^. However, the study results are often contentious or inconclusive because of the relatively small sample sizes or genuine heterogeneity. The reports of EMT-TF expression in BC and its association with prognosis are limited and controversial. Recent attention has focused on targeting factors upstream of EMT-TFs such as TWIST1, SNAIL1, and SLUG and understanding the role of hormone receptors in BC, may help the development of effective therapeutic interventions for patients with metastasis^18^,^19^.

The present meta-analysis, based on the published literatures, was aimed to investigate the relationship between EMT-TFs in MBC patients and major clinic pathological features, including the expression of estrogen receptor (ER), progesterone receptor (PR) and HER2.

## Results

### Literature search

**Figure 1:**
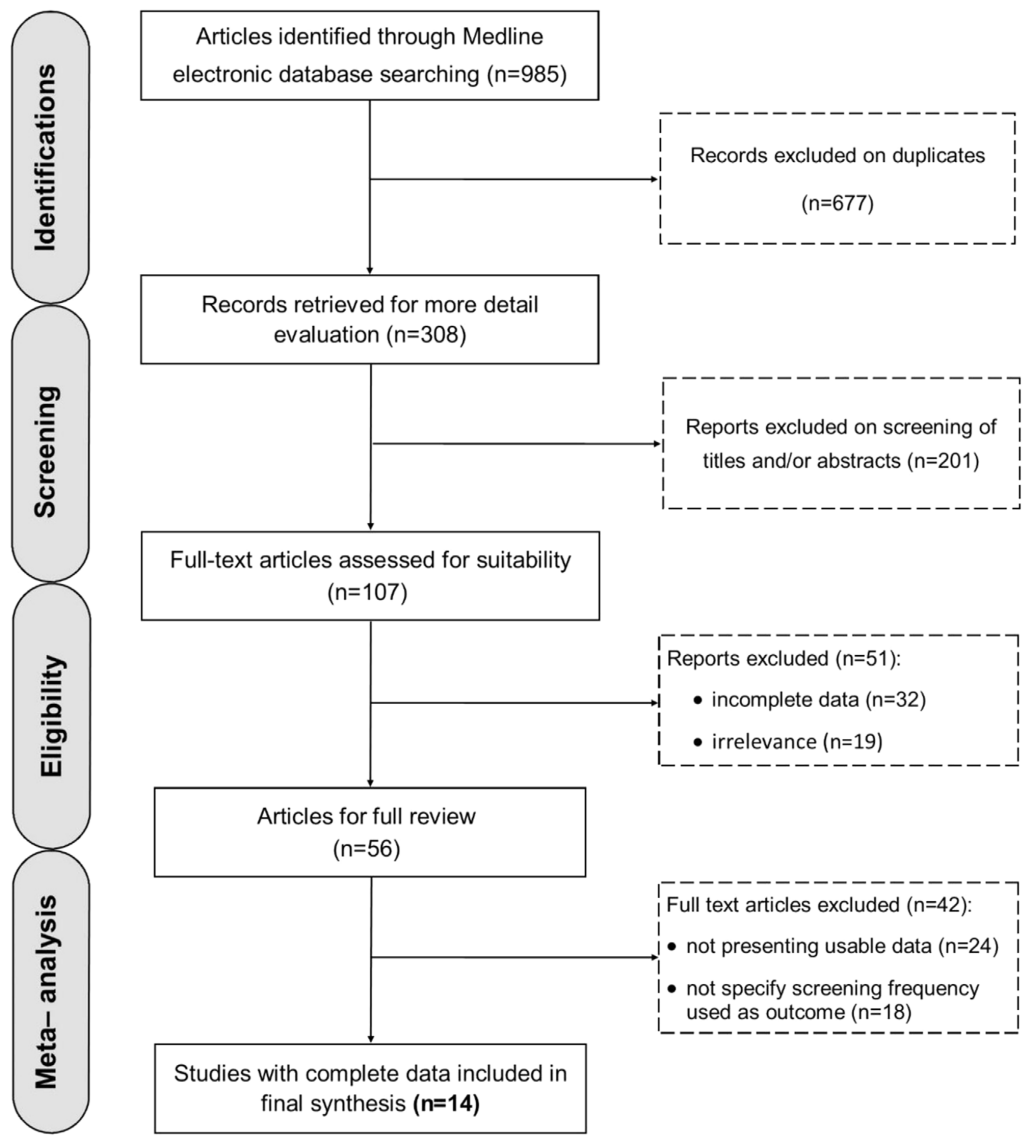
Schematic flow diagram for selection of the included studies in the meta-analysis.

As shown in Fig. 1, the literature search yielded 985 potentially relevant studies. Of these, 308 of the studies eligible for inclusion were confirmed with the initial search strategy. Next, 201 studies were excluded for unrelated titles and abstracts, and 107 full-text articles were assessed for suitability. However, 56 studies were precluded for obvious irrelevance, and 42 studies were precluded for insufficient data. A flow diagram describing the selection of the eligible trials and exclusion criteria is shown in Fig. 1.

### Study and patient characteristics

By reviewing the full-text of the remaining 56 articles, 14 studies were finally included in our meta-analysis and were distributed into 5 studies (29.40%) with 789 cases (20%) for TWIST1^13^,^19^,^20^,^21^,^22^, 8 studies (47.06%) with 2,512 cases (63.69%) for SNAIL1^19^,^21^,^23^,^24^,^25^,^26^,^27^, 2 studies (11.77%) with 89 cases (2.27%) for ZEB1^21^,^28^, and 2 studies (11.77%) with 551 cases (14.04%) for SLUG^29^,^30^.

The variables from fourteen relevant studies are summarized in Table 1 according to the first author’s name, number and characteristics of the cases for each study, primary antibody used for immunohistochemistry (IHC), sample size, population, TNM status. All the studies were published after 2008. Notably, only five studies reported prognostic data, among which information regarding HRs was directly extracted from three studies^25^,^26^,^27^,^29^,^30^. The studies were conducted mostly in Asia (8 studies, 57.14%)^13^,^22^,^23^,^26^,^27^,^28^,^29^,^30^ and Europe (6 studies, 42.86%)^19^,^20^,^21^,^24^,^25^,^31^, without any studies with American or African populations.

A total of 3,218 patients were included in these studies, and the median trial sample size was 250 patients. Most of the studies used a combined evaluation of cytoplasmic and nuclear staining for the determination of the expression status. Various antibodies were used in the evaluation of EMT-TF expression, although more studies used antibodies from Abcam Co. (Abcam Ltd., Cambridge, UK) (7 studies, 50%)^13^,^19^,^20^,^23^,^24^,^27^,^29^. The cutoff for positive expression depended on the staining score, and method. Based on breast cancer staging from the American Cancer Society^32^, the tumor-node-metastasis (TNMs) stage of most of the analyzed patients was ΙΙΙ B/C (11 studies, 78.58%)^13^,^19^,^20^,^21^,^22^,^23^,^24^,^25^,^27^,^29^,^30^. All 14 studies reported data that allowed the calculation of the 3-year overall survival (OS) rate. Seven studies (50.0%) presented data that allowed for assessment of 5-year OS^19^,^21^,^23^,^25^,^27^,^29^,^31^. Among the groups demonstrating EMT-TF overexpression, TWIST1 had the highest positive expression (54.92%) followed by SLUG (52.5%), SNAIL1 (45.58%), and ZEB1 (32%). The median follow-up time of the 10 reported studies that was 174 months (range = 60–240 months) (Table 1).

### Meta-analysis results

#### Association of EMT-TF expression with MBC prognosis

**Figure 2:**
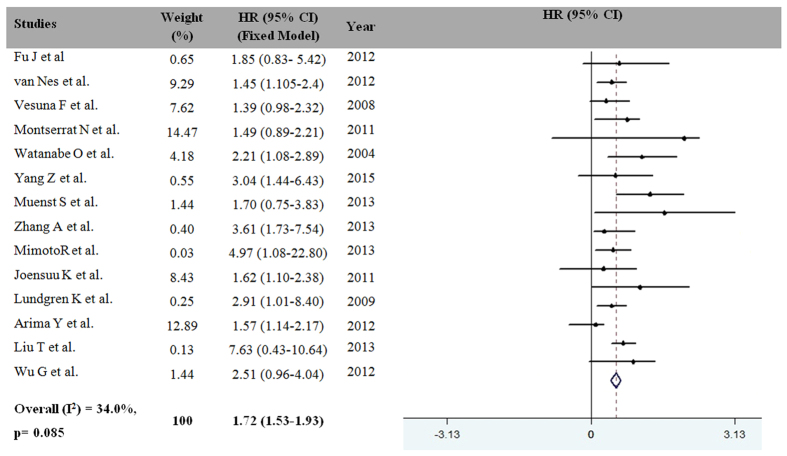
Meta-analysis results for EMT-TF expression and MBC risk. Weights are from the random-effects analysis. Abbreviations: HR, hazard ratio; CI, confidence interval.

The association between EMT-TF expression and the overall survival of MBC patients is shown in Fig. 2. This combined analysis of 14 studies indicated that EMT-TF expression was associated with a statistically significant 54% improvement in MBC compared with the control group (HR for death = 1.72, 95% CI = 1.53–1.93, P = 0.001). Consequently, the overexpression of EMT-TFs was a prognostic factor for MBC, and the heterogeneity of the overall prognosis was relatively low (I^2^ = 34.00%; P = 0.085) (Fig. 2). Therefore, we considered the fixed model to calculate the HRs for death.

#### Subgroup analysis

**Figure 3:**
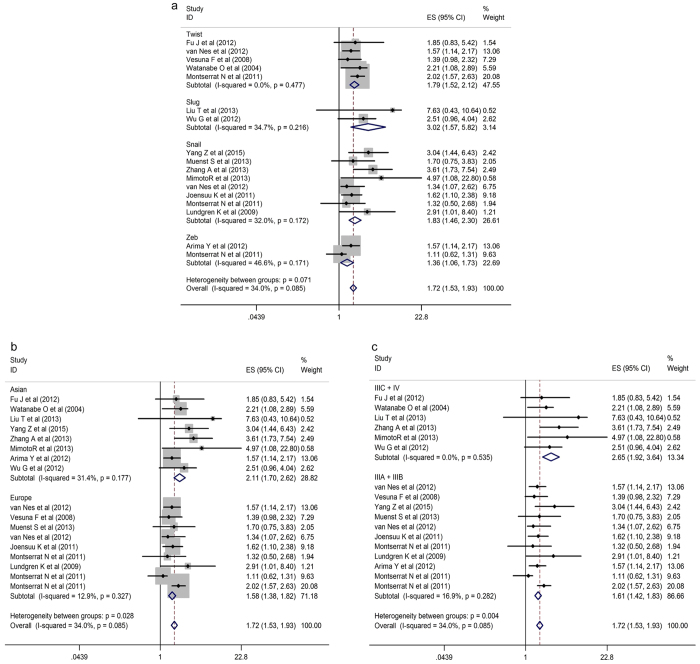
Forest plots showing that MBC was associated with the over-expression of EMT-TFs (**a**), race (**b**) and clinical stage (**c**). Weights are from the random-effects analysis. ES, effect size; CIs, confidence intervals.

Subgroup’s analysis was conducted regarding the type of EMT-TF, race, and TNM staging. As shown in Fig. 3, the HR for SLUG was significantly higher than that for TWIST1, ZEB1, and SNAIL1 (HR for death = 3.02, 95% CI = 1.57–5.82; P = 0.001). Additionally, the HRs for TWIST1 and SNAIL1 were 1.79 (95% CI = 1.52–2.12; P = 0.001) and 1.83 (95% CI = 1.46–2.30; P = 0.001), respectively. Strikingly, the HR for ZEB1 was less significantly different than that for other EMT-TFs (HR for death = 1.36, 95% CI = 1.06–1.73; P = 0.014) (Fig. 3a).

In the secondary sub-analysis, we evaluated the association between race and MBC risk. These results showed that the HR of Asian MBC patients was 2.11 (95% CI = 1.70–2.62; P = 0.001), while that of European MBC patients was 1.58 (95% CI = 1.38–1.82 P = 0.001) (Fig. 3b). However, EMT-TF overexpression was correlated with clinical stage (P ≤ 0.001) (IIIA + IIIB vs IIIC + IV, HR for death = 1.61, 95% CI  = 1.42–1.83 vs HR = 2.62 95% CI = 1.92–3.64, respectively), indicating that EMT-TF might have an association with advanced stages of MBC. Thus, these results suggest that EMT-TF potency contributes to cancer development and progression (Fig. 3c).

#### Publication bias and sensitivity analysis

**Figure 4:**
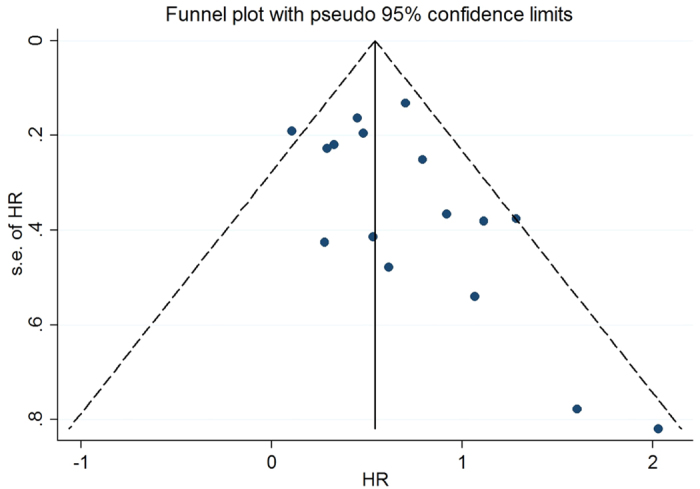
Funnel plot analysis to detect publication bias. Each point represents a single study for the specified association, the vertical axis represents the standard error of the logarithmic HR, and the horizontal axis represents the HR limits. HR, hazard ratio.

Investigations of publication bias and sensitivity were analyzed in the included literatures, including the overall HR with MBC, were carried out repeatedly by precluding one study at a time (Fig. 4)^33^. As shown in Fig. 4, the shape of the funnel plot and Egger’s test provided no statistical evidence for publication bias (t = 1.70, P = 0.11). Therefore, the lack of a noticeable publication bias influencing overall results in this meta-analysis showed that estimates before and after the deletion of each study were the same (data not shown), signifying that our meta-analysis results were stable and credible.

## Discussion

The current interests are in TWIST1, SNAIL1, and notably SLUG overexpression as potential prognostic markers for MBC because many experimental studies have linked SLUG, SNAIL1, and TWIST1 expression with worsening survival in cancers such as esophageal, oral, lung, ovarian and cervical cancers^8^,^34^,^35^,^36^,^37^,^38^,^39^,^40^. Thus TWIST1, SNAIL1, and especially SLUG expression might play a key role in the aggregations of MBC treatment as well as improve the follow-up plans in Asian MBC patients.

Although the association of nuclear TFs of EMT with the tumor metastatic process has been explored for several years, the available data have not been comprehensively analyzed until now.

In this study, we conducted a meta-analysis from the published data concerning the expression of TWIST1, ZEB1, SLUG, and SNAIL1 in MBC and their association with survival for studies that were evaluated by IHC. As expected, the pooled HR results showed that the expression of SNAIL1, SLUG and TWIST1 was associated with worsening survival. For the first time, the summary estimates support the hypothesis that TWIST1, SNAIL1 and SLUG expression is associated with worsening survival in MBC, in which SLUG might serve as the most significant prognostic marker for BC. Therefore, this study provided compelling evidence that the overexpression of TWIST1, SNAIL1, and SLUG1 individually or in combination may contribute to the progression of MBC, and further study could provide useful guidelines for physicians to improve follow-up plans for Asian MBC patients.

Several published meta-analyses have reported the relationship between EMT-TF expression and BC. For example, TWIST1 expression is associated with poor prognosis in patients with ER+ breast cancer^41^ and primary breast cancer^42^. The Zhang P. *et al*. meta-analysis results suggested that TWIST1 and SNAIL1 were prognostic factors for BC, with the pooled HR values of 2.07 (95% CI = 1.63–2.68) and 1.63 (95% CI = 1.33–1.99), respectively, indicating that TWIST1 may be a favorable prognosticative indicator of poor prognosis in MBC^39^. By contrast, Soini Y. *et al*. addressed that ZEB1 and TWIST1 are important transcription factors in EMT-transformed neoplastic cells and stromal fibroblastic cells^43^. The sample size, different inclusion and exclusion factors, and methods that assess the EMT-TF expression are the primary causes of this discrepancy. Furthermore, the relationship between EMT-TF overexpression and the clinical characterization of cancer had not been evaluated. In the present meta-analysis, the HR for SLUG overexpression-related death was considerably higher than those for SNAIL1 and TWIST1 (approximately 1.5-fold). Strikingly, the HR for ZEB1 was substantially less different than those of other EMT-TFs (HR for death = 1.36, 95% CI = 1.06–1.73).

Morphologically, most of these factors are involved in the disruption of intracellular adhesion, cytoskeletal rearrangements and finally migration and invasiveness. The major triggering signals that were reported concerning the EMT process are growth factors and/or cytokine signaling factors (e.g., TGF-β, epidermal growth factor (EGF), hepatocyte growth factor (HGF) and fibroblast growth factor (FGF)), integrin-related ECM components, Wnt proteins (Notch), hypoxia, small-molecule compounds, reactive oxygen species (ROS), and mechanical stress^6^,^7^,^44^.

**Figure 5:**
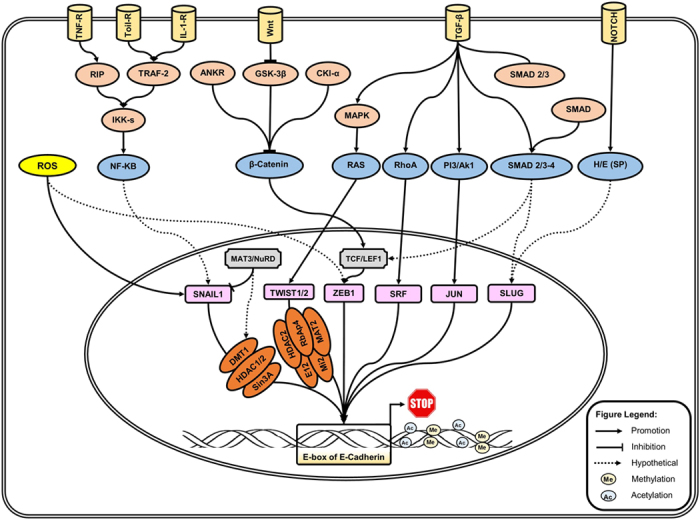
Major triggering signals and transcriptional regulators of EMT regulating E-cadherin. This schematic cartoon shows the major triggering of EMT-inducing signaling pathways and some of the points of intersection among the growth factors (TGF-β), cytokine tumor necrosis factor (TNF)-α, Wnt proteins (Notch) and ROS and other mechanical stress. The expression of EMT-induced transcription factors such as TWIST1, SLUG, SNAIL1, and ZEB1 lead to the repression of E-cadherin expression in the complex network (explained in detail in the main text of the manuscript). TNF-α (tumor necrosis factor alpha); IL-1 (interleukin-1); TGF-β (transforming growth factor beta); TRAF2 (TNF receptor-associated factor 2); ROS (reactive oxygen species), IKKs (Ikappa B kinases); GSK-3β (Glycogen synthase kinase 3 beta); MAPK (Mitogen-activated protein kinase); HDAC (histone deacetylase); DMT (DNA methyltransferase gene); SRF (serum response factor; Mi2/NuRD (Mi2/nucleosome remodeling and deacetylase protein complex); MTA2 (metastasis-associated protein 2); RbAp46 (Rb-associated protein 46).

EMT-inducing transcription factors such as TWIST1, SLUG, SNAIL1, ZEB1, E2-2 (TCF4) and E47 (TCF3) have recently emerged from these cascade pathways as nuclear transcription factors. The transcriptional regulators of EMT regulate E-cadherin and representative points of intersection between various EMT-inducing signaling pathways are shown in Fig. 5. As the schematic shows, the repression of E-cadherin by SNAIL1, TWIST1, or other repressors leads directly/indirectly to the expression of N-cadherin and Vimentin. Recently, it has been found that the hypermethylation and hypoacetylation H3/H4 of the E-cadherin promoter is associated with SNAIL1 overexpression and recruitment of the repressor complex formed by the corepressor Sin3A/histone deacetylase 1/2 (HDAC1/2)^45^,^46^. These interactions are dependent on the SNAG N-terminal domain of SNAIL1, which resulting in efficient repression of the E-cadherin promoter by the recruitment of a corepressor complex containing HDAC1/2 and Sin3A^46^. Additionally, TWIST1 expression is associated with the hypermethylation and hypoacetylation of the E-cadherin promoter in MBC, by recruiting TWIST1 plus Mi2/nucleosome remodeling and the deacetylase (Mi2/NuRD) protein complex^47^. The TWIST1/NuRD complex represses ERα expression in BC cells via the binding of TWIST1 to the E-box of the ER-α gene with E12 or TWIST2 interactions. TWIST1 forms a heterodimer with E12 to interact with the components of metastasis-associated protein 2 (MTA2) and Rb-associated protein 46 (RbAp46)^48^,^49^. The TWIST1/NuRD protein complex recruits the Mi2 and HDAC2 and to form the TWIST/Mi2/NuRD protein complex and reduce the acetylation and increase the methylation of ER-α^47^,^50^,^51^. Furthermore, the TWIST1/Mi2/NuRD protein complex represses ER-α expression in more than 85.7% of MDA-MB-435 cells, subsequently decreasing histone H3K9 acetylation, increasing histone H3K9 methylation, repressing the human *ESR1* gene encoding ERα, and suppresses E-cadherin expression to promote EMT and the metastasis of breast cancer^13^,^51^,^52^. These observations potentially suggest that the knockdown of TWIST1, MTA2 or RbAp46 plays a major role in the inhibition of MBC^53^ (Fig. 5).

Moreover, SNAIL1 and TWIST1 expression levels are positively correlated with increased micro-vessel density in cancers, where TWIST1 expression is positively correlated with cancer angiogenesis and invasiveness^54^. It is known that TWIST1 can stimulate angiogenesis feasibly through the up-regulation of MMPs (mostly MMP-9), Vimentin and N-cadherin^54^,^55^. Unfortunately, comparing with TWIST1 and SNAIL1, the functional characteristics of SLUG have been less studied. Further studies are required to explore the clinical utility of other EMT-TFs in MBC.

For the first time, our results demonstrated that the HRs of Asian MBC patients are greater than those of European MBC patients (approximately 1.3-fold). Life-style factors such as alcohol exposure, tobacco inhalation, nutrition, and environment might have synergistic effects on EMT-TF expression^54^,^56^. For example, it is well known that benzo(a)pyrene in tobacco modulates TWIST1 and SNAIL1 expression and promotes the migration and invasion of BC^54^,^56^. Increased smoking and/or lifestyle in developing countries could be a justification for the findings. Consequently, future studies are warranted to explore the interactions of confounding factors with EMT-TFs on BC risk.

The prognosis of MBC patients is quite different. In this study, the TNM stage of most of the analyzed patients was ΙΙΙ B/C stage (11 studies, 78.58%). The meta-regression analysis results showed that clinical tumor stage was the main source of heterogeneity. When these 11 studies were omitted, the heterogeneity was reduced, strongly establishing that patients with MBC should be analyzed separately to achieve additional optimal results^57^,^58^.

The above findings might help to explain why EMT-TFs might contribute to BC progression and the identification of novel therapeutic targets of MBC progression. However, it should be noted that many upcoming studies are needed to illustrate the exact mechanisms because the published evidence is limited. Few studies have demonstrated the correlations between EMT-TF gene or mRNA expression and prognosis.

We should note that there are some limitations in this study. First, we only included the studies in the English language, and published studies in other languages, particularly Chinese and Russian, were not included. Inevitably, the most important limitation lies in the evaluation of gene expression techniques. As previously mentioned, the eligibility criteria were the assessment of TWIST1, SNAIL1, SLUG, and ZEB1 expression by IHC, which utilizes different individual primary antibodies and antibody dilutions; the IHC staining protocol might also affect the IHC sensitivity. Importantly, the cut-off uniform definition of EMT-TFs performed was different for each study, possibly affecting the precision of the estimate. Other evaluation techniques such as microarray, fluorescence *in situ* hybridization, real-time polymerase chain reaction (RT-PCR), and whole-exome sequencing are used at a very low frequency and provide limited information^18^,^39^,^40^. Certainly, the results of this meta-analysis should be interpreted cautiously because there might be underlying heterogeneity. In addition, there was no homogeneous distribution among the population in our study.

Furthermore, most of the published studies did not disclose information on the patients’ preoperative or postoperative treatments. The type of adjuvant therapy each patient received may also affect the prognosis of the patients. All this insufficient information could contribute to additional inconsistencies and create potential selection bias. Of course, our current data need to be substantiated by adequate prospective studies. Thus, the outcomes might only represent a typical part of societies worldwide, partially influencing the inter-study heterogeneity.

Despite these limitations, the data of the present meta-analysis suggest that TWIST1, SNAIL1 and SLUG overexpression might play critical roles in the development of MBC and improves the follow-up plans in Asian MBC patients. In addition, we specially evaluated the risk of high SLUG, SNAIL1, and TWIST1 levels and the worsening survival in MBC patients. Taken together, analyzing specific factors linked to MBC will not only enhance our understanding of the roles of EMT-TFs but also provide effective therapeutic strategies to target MBC.

## Materials and Methods

### Search strategy and selection studies

A comprehensive systematic search from the literatures published in English was carried out by querying the MEDLINE electronic database such as PubMed, ISI Web of Science, and Embase to identify all the relevant studies prior to November 1, 2015; no lower date limit was used. The search string was conducted by deriving the main heading term based on the research question, such as: “SNAIL1 and breast cancer” or “SNAIL1 metastatic breast cancer” or “SNAIL1 and metastatic breast cancer survival” or “SNAIL1 and breast cancer and prognosis” or “SNAIL1 and breast cancer and outcome.” In addition, we repeated this search for “TWIST1,” “SLUG,” and “SNAIL1” and another key EMT-TFs (for example “ZEB1” and breast cancer survival) to recognize additional studies. Alternative spelling and synonyms were incorporated using the Boolean “OR” term, and the main terms were linked using the Boolean “AND” Term.

### Exclusion criteria

Exclusion criteria in this meta-analysis were as follows: (*i*) reviews, case-controls, letters or experiments on cell lines and animal models; (*ii*) studies evaluating the gene expression of EMT-TFs measured by RT-PCR, microarray, or fluorescence *in situ* hybridization; (*iii*) studies investigating the relationship of single-nucleotide polymorphisms (SNPs) in BC.

The citation lists of the retrieved articles were manually screened to ensure the sensitivity of the search strategy. Eligibility criteria were the assessment of TWIST1, SNAIL1, SLUG, and ZEB1 expression by IHC, availability of survival data for at least 3 years, and publication in English.

When an individual author published several articles with data obtained from the same patient population, only the newest or most informative article was selected. Study selection was based on the association of each of EMT-TF with survival.

### Data collection and study assessment

All the selected articles were analyzed by two independent investigators (SI and JC) according to the PICO principle^59^, and any inconsistency or disagreement in the research process was resolved through debate and consultations. If a consensus could not be reached, a third partner was consulted. The electronic investigation was supplemented by a hand-search of relevant articles from the reference lists to ensure that all the studies could be identified. Finally, the following details were extracted: first author’s name, year of publication, geographical location, sample size of the different histological categories of BC, grade of BC, stage of BC, and ER, PR, HER2 status, follow-up time and antibody used for the IHC, methods and scores for the assessment, cut-off for considering TWIST1, SNAIL1, SLUG, and ZEB1 as positive expression, and hazard ratios (HRs) with corresponding 95% confidence intervals (CIs). If the above information was not mentioned in the original study, the item was treated as “not reported (NR).”

### Statistical analysis

The meta-analysis was performed using Manager Software version 5.2 (software updates; The Nordic Cochrane Centre, Copenhagen, Denmark). Data combining was performed by RevMan version 5.2 (free software downloaded from http://www.cochrane.org, The Cochrane Collaboration, The Nordic Cochrane Centre, Copenhagen, Denmark, 2012). Hardy-Weinberg equilibrium (HWE) was checked by χ^2^ test. The chi-square-based Q-test was applied to testify between-study heterogeneity. HR was calculated by the fixed-effects model with P (heterogeneity) >0.05, as a secondary analysis^60^. Otherwise, the random-effects model was used. According to our hypothesis and inclusion criteria, HR >1 implies poor prognosis for a high TWIST1 level and pooled HRs with 95% CIs were used to assess the relationship between EMT-TFs and MBC susceptibility. The HRs and 95% CIs were calculated by Tierney’s methods if the data were not reported in the original study^61^. Subgroup analysis was conducted for the association between the type of EMT-TF and race. Publication bias was evaluated by Begg’s funnel plots^62^ and Egger’s regression test^63^. A value of “Pr > |z|” less than 0.05 was considered to indicate potential publication bias^63^,^64^. Additionally, we also conducted a sensitivity analysis by precluding a single study at a time to observe whether the pooled HRs changed. All the reported P values were two-sided, and P < 0.05 was considered to indicate statistical significance. Statistical analyses were carried out using STATA version 11.0 (Stata Corporation, College Station, TX, USA).

## Additional Information

**How to cite this article**: Imani, S. *et al*. Prognostic Value of EMT-inducing Transcription Factors (EMT-TFs) in Metastatic Breast Cancer: A Systematic Review and Meta-analysis. *Sci. Rep.* **6**, 28587; doi: 10.1038/srep28587 (2016).

## Figures and Tables

**Table 1:** Main results of the selected studies.

EMT-TF	Author (Ref.)	Year	SS	PA	Pop.	TNM	TFP (%)	FT (M)
TWIST1	Fu J. *et al*.^13^	2012	28	ab 50887, Abcam, UK	CN	ΙΙΙ C	61	NR
	van Nes *et al*.^19^	2012	575	ab5088, Abcam, UK	NL	ΙΙΙ B	50	240
	Vesuna F. *et al*.^20^	2008	87	Abcam, UK	NL	ΙΙΙ B	65	NR
	Montserrat N. *et al*.^21^	2011	76	–	ES	ΙΙΙ B	29	200
	Watanabe O. *et al*.^22^	2004	23	Santa Cruz, CA, USA	JP	Ш B/C	69.6	NR
SNAIL1	Yang Z. *et al*.^23^	2015	125	Abcam, UK	CN	ΙΙΙ B	38.4	120
	Muenst S. *et al*.^25^	2013	1043	Santa Cruz, CA, USA	CH	ΙΙΙ B	49.4	120
	Zhang A. *et al*.^26^	2013	60	Santa Cruz, CA, USA	CN	Ш C/IV	47	NR
	Mimoto R. *et al*.^27^	2013	61	Abcam, UK	JP	Ш C	47.6	120
	van Nes. *et al*.^19^	2012	575	ab27568, Abcam, UK	NL	Ш B	54.5	240
	Joensuu K. *et al*.^24^	2011	73	ab17732, Abcam, UK	FI	ΙΙΙ B	75	NR
	Montserrat N. *et al*.^21^	2011	75	–	ES	ΙΙΙ B	29	200
	Lundgren K. *et al*.^31^	2009	500	CST, Beverly, MA, USA	SE	ΙΙΙ A	23.7	240
ZEB1	Arima Y. *et al*.^28^	2012	14	Vector Lab, CA, USA	JP	ΙΙΙ A/B	45	NR
	Montserrat N. *et al*.^21^	2011	75	–	ES	ΙΙΙ B	19	200
SLUG	Liu T. *et al*.^29^	2013	441	Abcam, UK	CN	ΙΙΙ C	61	60
	Wu G. *et al*.^30^	2012	113	CST, Beverly, MA, USA	CN	ΙΙΙ C	44	NR

Abbreviations: EMT-TFs, EMT-inducing transcription factors; SS, sample size; PA, primary antibody used for IHC; Pop., populations; TNM, tumor-node-metastasis; TFP, EMT-TF positive; FT, flow time; M, months; NR, not reported.
